# Priorisierung von Beschäftigten einer medizinischen Einrichtung der Maximalversorgung bei der Impfung gegen COVID-19: Herausforderungen und Lösungsansätze: Ein Diskussionsbeitrag aus der Praxis

**DOI:** 10.1007/s00481-022-00682-1

**Published:** 2022-01-18

**Authors:** Katharina Beier, Alfred Simon, Michael P. Schön

**Affiliations:** 1 Institut für Ethik und Geschichte der Medizin, Universitätsmedizin Göttingen, Humboldtallee 36, 37073 Göttingen, Deutschland; 2 Akademie für Ethik in der Medizin e. V., Göttingen, Deutschland; 3 Klinik für Dermatologie, Venerologie und Allergologie, Universitätsmedizin Göttingen, Göttingen, Deutschland

Einrichtungen der medizinischen Maximalversorgung, wie Universitätsklinika, leisten einen zentralen Beitrag zur Beherrschung der COVID-19-Pandemie. Um den Versorgungsauftrag zu sichern und Ansteckungen innerhalb der Einrichtungen zu verhindern, wurde medizinischem Personal mit hohem Expositionsrisiko und/oder Kontakt zu vulnerablen Menschen Vorrang bei der Verteilung der zunächst knappen Impfstoffe eingeräumt (vgl. STIKO 2020; BMG 2021). Einrichtungen mit mehreren Tausend Beschäftigten in verschiedenen Einsatzbereichen, hochgradiger Arbeitsteilung und individuell heterogenen Gefährdungslagen mussten einerseits die Impflogistik bei angespannter Versorgungslage aufbauen, andererseits auch Strukturen für nachvollziehbare, transparente und ethisch begründete Entscheidungen zur Impfpriorisierung etablieren. Solche Entscheidungsfragen stellten sich, weil in den Empfehlungen/Anordnungen der Makroebene nicht alle Beschäftigtengruppen sowie deren tatsächliche Gefährdungslagen erfasst wurden und im dynamischen Prozess der Pandemie immer wieder situationsspezifische Anpassungen an neue Erkenntnisse sowie die schwankende Verfügbarkeit der Vakzine erforderlich waren. Am Beispiel des „Mikrokosmos“ der Universitätsmedizin Göttingen (UMG) mit ca. 8000 Beschäftigten und 4000 Studierenden beschreiben wir das Vorgehen für einen ethisch reflektierten Umgang mit den Verteilungsfragen und diskutieren Implikationen für vergleichbare zukünftige Mangelsituationen.

## Rahmenbedingungen für die Impfpriorisierung

Die erste Empfehlung zur Priorisierung der Schutzimpfung gegen SARS-CoV‑2 erfolgte durch die Ständige Impfkommission (STIKO) im Dezember 2020 und die Festlegung des Rechtsrahmens für den Anspruch auf eine Schutzimpfung durch das Bundesministerium für Gesundheit (BMG) im Februar 2021 (seitdem mehrfach aktualisiert). Vor der für Januar 2021 erwarteten ersten Impfstofflieferung an die UMG setzte der Vorstand im Dezember 2020 eine *Impf-Task-Force* ein. Auf der Grundlage der STIKO-Empfehlung erarbeitete diese zusammen mit Vertreter*innen des Klinischen Ethikkomitees (KEK) eine sechsstufige, verschiedene Tätigkeitsfelder- und Beschäftigtengruppen berücksichtigende Priorisierungsliste. Da zu Beginn nicht für alle Beschäftigten mit besonders hohem Expositionsrisiko oder engem Kontakt zu vulnerablen Personen (STIKO-Stufe 1; § 2 der Corona-Impfverordnung) ausreichend Impfstoff zur Verfügung stand, erfolgte insbesondere für Stufe 1 eine Subpriorisierung dahingehend, dass zwischen Mitarbeitenden mit besonders hohem Expositionsrisiko (Stufe 1a: Notaufnahme, Rettungsdienst, COVID-19-Stationen), Mitarbeitenden mit engem Kontakt zu besonders vulnerablen Patient*innen (Stufe 1b: Intensivstationen, Hämatologie/Onkologie, Geriatrie) und Mitarbeitenden mit engem Kontakt zu vulnerablen Patient*innen (Stufe 1c: Palliativmedizin, Strahlentherapie, Pädiatrie) unterschieden wurde (siehe die vollständige Empfehlung zur Priorisierung im Zusatzmaterial zu diesem Artikel).

## Ethik Board

**Figure 1:**
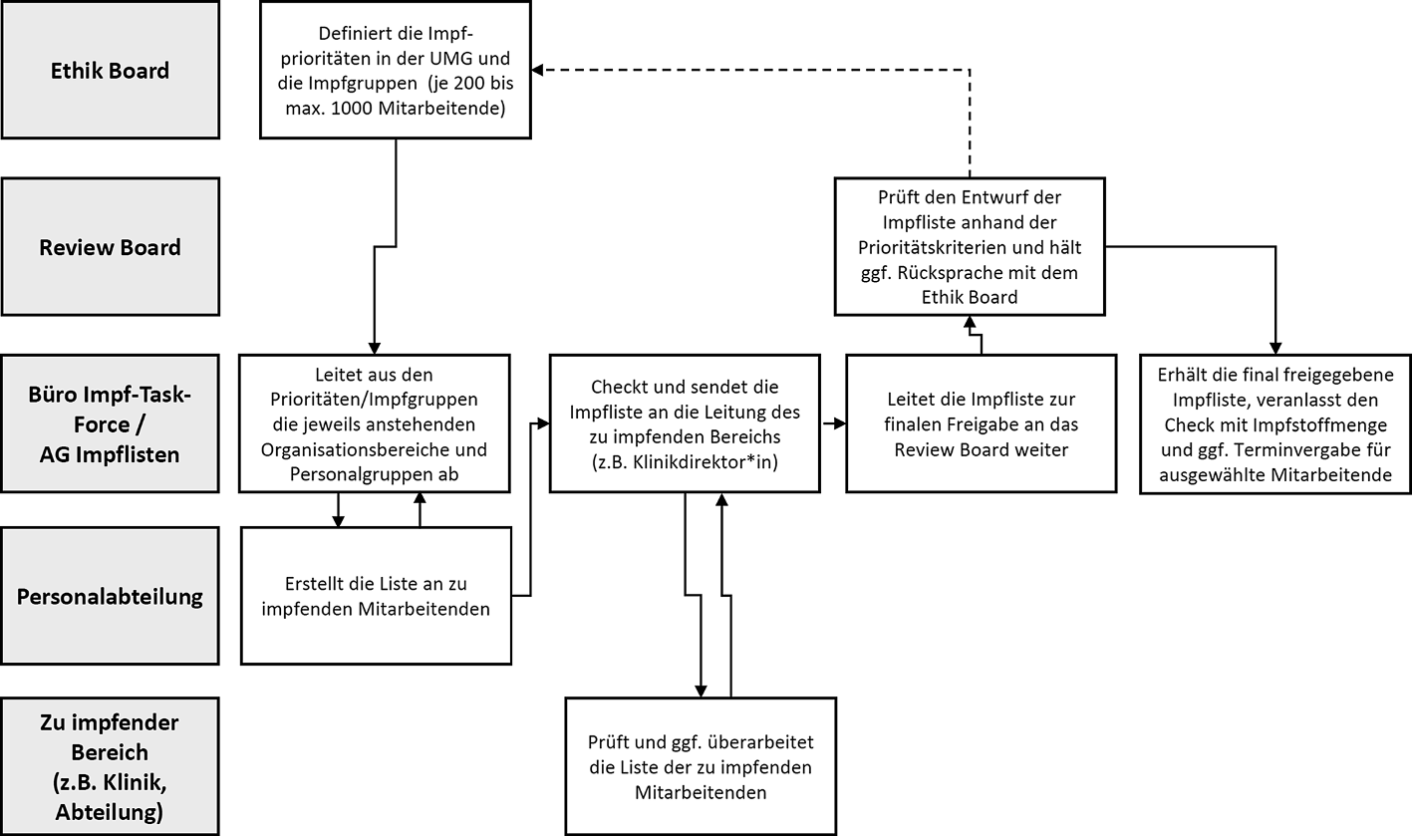

Die Erfahrung bei der Impfung der ersten ca. 1000 Beschäftigten (besonders aus COVID-19-Bereichen und Notfallversorgung) zeigte, dass die Umsetzung dieser Priorisierungsliste hohe Anforderungen an interne Organisations- und Kommunikationsstrukturen stellte und Unschärfen bezüglich der Zuordnung aufwies. So barg der Umstand, dass nicht allen Mitarbeitenden gleichzeitig ein Impfangebot gemacht werden konnte, erhebliches Konfliktpotential – etwa, weil Beschäftigte(ngruppen) sich hinsichtlich der ihnen zugewiesenen Priorität untereinander verglichen und ihre spezifische Gefährdungslage nicht angemessen abgebildet sahen. Zudem bestanden innerhalb einzelner Einrichtungen durchaus individuelle Unterschiede bezüglich der Gefährdung. Um diesen teils berechtigten Bedenken, Beschwerden und Ansprüchen Rechnung zu tragen und den Betriebsfrieden zu wahren, wurde im Februar 2021 als Teil der Impf-Task-Force ein interdisziplinäres *Ethik Board* mit Vertreter*innen aus (Intensiv‑)Medizin, Pflege, Forschung, technischem Personal, KEK und Personalrat eingesetzt. Für die Koordination wurde eine medizinethisch geschulte wissenschaftliche Referentin angestellt. Aufgabe des Ethik Boards war es, die Priorisierung der Mitarbeitenden(gruppen) im Lichte der Kriterien der STIKO/BMG-Verordnung zu prüfen und Grundsatzempfehlungen für eine der Arbeitsrealität Rechnung tragende Eingruppierung auszusprechen. Anfragen an das Ethik Board konnten sowohl von Beschäftigten(gruppen) als auch durch Gremien der Impf-Task-Force gerichtet werden. Die Empfehlungen des Ethik Boards wurden nach Annahme durch den Vorstand durch das *Review Board*, dem die Erstellung der Impflisten oblag, umgesetzt. Dies gewährleistete die gegenseitige Kontrolle verschiedener Gremien (Abb. 1).

## Partizipation der Einrichtungen

In Reaktion auf die unter verschiedenen Beschäftigten(gruppen) umstrittene Priorisierungsliste führte das Ethik Board eine transparent kommunizierte, kriteriengeleitete Abfrage bei den Einrichtungsleitungen durch, um Mitarbeitende zu identifizieren, deren spezifische Gefährdungslage in der pauschalen Eingruppierung nicht angemessen abgebildet worden war. Dabei wurde die höchste Priorität (1a) für diejenigen angenommen, die i) unvermeidbar direkten Kontakt zu Personen mit diagnostizierter COVID-19-Erkrankung haben, ii) aerosolgenerierende Tätigkeiten an Patient*innen durchführen bzw. bei diesen unvermeidbar anwesend sind oder iii) geplanten und durch die Art der Behandlung unvermeidbar ungeschützten, engen Kontakt zu Personen mit unbekanntem Infektionsstatus haben. Im Ergebnis zeigte sich – insbesondere unter Berücksichtigung des Kriteriums iii –, dass deutlich mehr Beschäftigte in die höchste Prioritätsstufe (1a) einzuordnen waren als ursprünglich vorgesehen (etwa in Ambulanzen mehrerer Fachbereiche). Auch wenn sich damit das Verteilungsproblem praktisch zunächst verschärfte, führte die explizite, an einheitlichen Kriterien ausgerichtete Anerkennung der höchsten Impfpriorität für bislang niedriger priorisierte Personen zu besserer Akzeptanz insgesamt sowie zu mehr Verständnis für die mangelbedingten Wartezeiten bis zum Erhalt eines Impfangebots.

Um besonderen Arbeitsumständen einzelner Beschäftigter Rechnung zu tragen, konnten die Einrichtungsleitungen zudem Personen benennen, deren Ausfall nicht hätte kompensiert werden können, so dass die Funktionsfähigkeit eines wesentlichen Bereichs gefährdet gewesen wäre (kritische Funktionen). Um den exzessiven Gebrauch dieses Kriteriums zu vermeiden, musste die Einordnung explizit begründet werden. Sie konnte allenfalls zur Eingruppierung in Prioritätsstufe 2 führen.

## Empfehlungen des Ethik Boards

Über die Feinjustierung der Priorisierungsliste hinaus erarbeitete das Ethik Board – gebündelt in 12 Grundsatzempfehlungen – Stellungnahmen zu insgesamt 30 Anfragen. Inhaltlich lassen sich die Fragestellungen im Wesentlichen vier Kategorien zuordnen:

### In welcher Reihenfolge impfen?

Am häufigsten wurden Empfehlungen hinsichtlich der Impfpriorität für Beschäftigte, deren Eingruppierung sich aus den definierten Prioritäten nicht unmittelbar ableiten ließ, angefragt. Solche Fragen stellten sich etwa für Mitarbeitende des Giftnotrufs, die im Studienzentrum tätigen Study Nurses, Beschäftigte, die zwar nicht direkt mit infizierten Personen, aber mit deren potenziell infektiösen Materialien Kontakt haben, Therapeut*innen mit heterogenen Einsatzbereichen sowie auch Studierende, Schüler*innen und Auszubildende. Weitere Einordnungsfragen ergaben sich mit Blick auf ehrenamtlich für die UMG tätige Personen, wie Seelsorger*innen, Hospizmitarbeiter*innen, „Grüne Damen“ oder externe Mitglieder des KEK. Leitend für die Empfehlungen zu diesen Fragen waren weder Berufsgruppenzugehörigkeit noch Beschäftigungsstatus, sondern allein die Einschätzung der konkreten Gefährdungslage der betreffenden Personen. Um zu konsistenten und rechtskonformen Empfehlungen zu gelangen, prüfte das Ethik Board zudem, inwiefern für die fraglichen Personen analoge Gefährdungslagen zu den durch die BMG-Verordnung explizit benannten Gruppen anzunehmen waren. Entsprechend wurde etwa für die „Grünen Damen“, die bei ihrem altruistischen Engagement mit vielen Patient*innen in Kontakt kommen, – nicht zuletzt im Sinne einer reziprok verstandenen Solidarität – ein vergleichbar hohes Expositionsrisiko wie für die in Stufe 2 fallenden Mitarbeitenden angenommen.

### Antizipierte vs. tatsächliche Gefährdung

Weitere Anfragen bezogen sich darauf, ob bzw. wann (noch) geimpft werden sollte. So galt es zu klären, wann Rotationsärzt*innen und aus der Elternzeit zurückkehrende Personen, die in einem Bereich mit hoher Impfpriorität eingesetzt werden sollten, ein Impfangebot erhalten sollten. Zunächst sprach sich das Ethik Board dafür aus, diesen Personen erst dann ein Impfangebot zu machen, wenn sie tatsächlich im betreffenden Bereich eingesetzt sind, und in der Zeit des noch fehlenden vollständigen Impfschutzes das Expositionsrisiko und/oder Kontakt zu vulnerablen Patient*innen durch andere Maßnahmen zu reduzieren. Dadurch sollten die knappen Ressourcen zum größtmöglichen Nutzen eingesetzt und zunächst die tatsächlich Exponierten geimpft werden. Bei zunehmender Verfügbarkeit von Impfstoff wurde die Empfehlung dahingehend revidiert, dass ein Impfangebot so frühzeitig erfolgen sollte, dass die betreffenden Mitarbeitenden ihren Einsatz mit vollständigem Impfschutz beginnen können.

### Personen, für die keine STIKO-Empfehlung vorliegt, und Fürsorgepflicht der Arbeitgeberin

Da es zur Impfung Schwangerer zunächst keine Empfehlung der STIKO gab, befasste sich das Ethik Board mit der Frage, ob sich diese auf eigenen Wunsch impfen lassen können sollen. Unter Berücksichtigung der Empfehlung der Deutschen Gesellschaft für Gynäkologie und Geburtshilfe (DGGG) und des Betriebsärztlichen Dienstes sprach sich das Ethik Board dafür aus, Schwangere auf eigenen Wunsch nach ausführlicher Beratung an der UMG zu impfen.

Ferner galt es zu klären, ob bei Beendigung des Beschäftigungsverhältnisses nach der Erstimpfung Anspruch auf die Zweitimpfung durch die ehemalige Arbeitgeberin besteht. Aus oben genannten Gründen verneinte das Ethik Board auch hier zunächst einen solchen Anspruch, empfahl jedoch die Berücksichtigung von Härtefällen, in denen die Zweitimpfung im entsprechenden Zeitrahmen sonst nachweislich nicht möglich wäre, und stellte die Überarbeitung der Empfehlung bei entspannter Versorgungslage in Aussicht.

### Mit welchem Impfstoff?

Aufgrund sich mehrender Hinweise auf Nebenwirkungen des AstraZeneca-Impfstoffs kam es auch unter medizinischem Personal zu (oft emotional vorgetragenen) Einwänden – sowohl gegen den Einsatz dieses Vakzins als auch den Verzicht darauf. Das Ethik Board befasste sich daher noch vor entsprechender Änderung der STIKO-Empfehlung mit der Frage, ob Mitarbeitende das Recht haben sollten, den Impfstoff frei zu wählen. Es sprach sich – auch unter Berufung auf gesetzliche Grundlagen und solange Lieferungen bestimmter Impfstoffe nicht plan- oder vorhersehbar waren – zunächst gegen einen Anspruch auf einen bestimmten Impfstoff aus. Eine an der Verfügbarkeit eines bestimmten Impfstoffs ausgerichtete strategische Impfanmeldung sollte nicht befördert werden.

## Resümee

Fragen der Priorisierung zeitnah durch ein verschiedene Disziplinen und Statusgruppen vereinendes Gremium zu beantworten, erwies sich als sinnvoll, um die von STIKO und BMG formulierten Impfziele an der UMG bestmöglich umzusetzen und individuelle Gefährdungslagen objektiv und transparent in eine gerechte (und als gerecht empfundene) Rangfolge für ein Impfangebot zu übersetzen. Die Festlegung von Verantwortlichkeiten innerhalb der Impf-Task-Force sowie die kontinuierliche Rückkopplung zwischen verschiedenen Gremien trugen dazu bei, dass Entscheidungen an konsistenten Kriterien ausgerichtet und in wechselseitiger Kontrolle erfolgten. Die Empfehlungen des Ethik Boards wurden samt ausführlicher ethischer Begründung dokumentiert und bei Relevanz für einen breiten Kreis auch über den Corona-Krisenstab kommuniziert. Ein weiterer Vorteil bestand darin, dass auf dynamische Entwicklungen, etwa bezüglich der Verfügbarkeit oder Indikation von Impfstoffen bei bestimmten Personen, flexibel reagiert werden konnte. So passte das Ethik Board seine Empfehlungen mehrfach an sich ändernde Rahmenbedingungen an.

Der Aufbau entsprechender Kommunikations- und Entscheidungswege brachte eine Reihe von Herausforderungen mit sich. So mussten sich die aus verschiedenen Bereichen in die Gremien der Impf-Task-Force entsandten Mitglieder zunächst in ihre Aufgaben einfinden und Routine in der Zusammenarbeit entwickeln. Damit gingen Herausforderungen für die Kommunikation mit den Beschäftigten einher. So war das komplexe Gefüge der Impf-Task-Force nicht leicht verständlich, weshalb Anfragen von Beschäftigten manchmal parallel an mehrere oder das falsche Gremien gerichtet wurden. Dies hatte einen erhöhten Kommunikationsaufwand zur Folge. So stand das Ethik Board in kontinuierlichem Austausch mit dem Büro der Impf-Task-Force, um Einzelanfragen jenseits ethischer Grundsatzfragen an dieses zurückzuverweisen.

Der Umgang mit knappen Ressourcen ist gerade in Einrichtungen der Maximalversorgung mit sehr vielen unterschiedlichen Bereichen organisationsethisch komplex. Um für derartige Situationen auch in Zukunft gerüstet zu sein, sollten Kommunikations- und Entscheidungswege so vorgeplant sein, dass sie im Bedarfsfall schnell verfügbar sind. Die Einsetzung eines interdisziplinären Ethik-Gremiums, eingebettet in ein System von „*checks and balances*“, kann dazu beitragen, dass Priorisierungen oder auch Rationierungen ethisch begründet und unter Berücksichtigung des konkreten Kontextes erfolgen und somit innerhalb der Einrichtung die notwendige Akzeptanz finden.

## Supplementary Information




