# Supplementary material for: Priorisierung von Beschäftigten einer medizinischen Einrichtung der Maximalversorgung bei der Impfung gegen COVID-19: Herausforderungen und Lösungsansätze: Ein Diskussionsbeitrag aus der Praxis
Source: Ethik Med. 2022 Jan 18;34(1):111–8. [Article in German] doi: 10.1007/s00481-022-00682-1 (PMC8764648; doi:10.1007/s00481-022-00682-1)
Supplement: Supplementary file 1 [file 481_2022_682_MOESM1_ESM.docx]

**Katharina Beier, Alfred Simon, Michael P. Schön: Priorisierung von Beschäftigten einer medizinischen Einrichtung der Maximalversorgung bei der Impfung gegen COVID-19: Herausforderungen und Lösungsansätze – Ein Diskussionsbeitrag aus der Praxis**

**Elektronisches Zusatzmaterial**

**Empfehlung der COVID-19 Impfung für die Mitarbeitenden der UMG (UMG Empfehlung)**

*Erarbeitet von der UMG-Impf-Task-Force in Zusammenarbeit mit dem Klinischen*

*Ethikkomitee der UMG*

| Stufe* | Mitarbeitende (Beispiel) |
| --- | --- |
| 1a | **Mitarbeitende mit besonders hohem Expositionsrisiko** (z.B. Notaufnahme, Rettungsdienst und COVID-19-Stationen) |
| 1b | **Mitarbeitende mit besonders engem Kontakt zu besonders vulnerablen Personen** (z.B. Intensivstationen, Hämatologie/Onkologie, Gynäkologie/Geburtshilfe, Geriatrie, Gerontopsychiatrie) |
| 1c | **Mitarbeitende mit engem Kontakt zu vulnerablen Personen**  (z.B. Palliativmedizin, Strahlentherapie, Pädiatrie) |
| 2 | **Mitarbeitende mit hohem Expositionsrisiko** (z.B. Hals-Nasen-Ohren-Heilkunde, Zahnkliniken, Psychiatrie, Internistische Kliniken und Kliniken mit vergleichbarem Risiko, Radiologie, Pflegepool, Studierende im Praktischen Jahr, Sicherheitsdienst der UMG) |
| 3 | **Mitarbeitende mit moderatem Expositionsrisiko** (weitere Mitarbeitende mit Patientenkontakt, z.B. Reinigungsdienst, UMG Gastronomie, Versorgungskoordinator*innen, Handwerker*innen im Technischen Gebäudemanagement, Medizinstudierende) |
| 4 | **Mitarbeitende mit niedrigem Expositionsrisiko** (weitere Mitarbeitende ohne Patientenkontakt, z.B. alle anderen Mitarbeitenden ohne Patientenkontakt aus den vorgenannten Bereichen, Labore, Verwaltung; technischer Dienst, Wäscherei, Forschungsbereich, Lehre, alle weiteren Medizin-Studierenden, alle weiteren UMG Facilities Beschäftigten) |

*Priorisierung innerhalb der Stufen absteigend nach Alter
